# Improving the diagnostic strategy for thyroid nodules: a combination of artificial intelligence-based computer-aided diagnosis system and shear wave elastography

**DOI:** 10.1007/s12020-024-04053-2

**Published:** 2024-10-07

**Authors:** Ziman Chen, Nonhlanhla Chambara, Xina Lo, Shirley Yuk Wah Liu, Simon Takadiyi Gunda, Xinyang Han, Michael Tin Cheung Ying

**Affiliations:** 1 Department of Health Technology and Informatics, The Hong Kong Polytechnic University, Kowloon, Hong Kong; 2 School of Healthcare Sciences, Cardiff University, Cardiff, UK; 3 Department of Surgery, North District Hospital, Sheung Shui, New Territories Hong Kong; 4 Department of Surgery, The Chinese University of Hong Kong, Prince of Wales Hospital, Shatin, New Territories Hong Kong

**Keywords:** Thyroid nodule, Computer-aided diagnosis, Shear wave elastography, Ultrasound

## Abstract

**Purpose:**

Thyroid nodules are highly prevalent in the general population, posing a clinical challenge in accurately distinguishing between benign and malignant cases. This study aimed to investigate the diagnostic performance of different strategies, utilizing a combination of a computer-aided diagnosis system (AmCAD) and shear wave elastography (SWE) imaging, to effectively differentiate benign and malignant thyroid nodules in ultrasonography.

**Methods:**

A total of 126 thyroid nodules with pathological confirmation were prospectively included in this study. The AmCAD was utilized to analyze the ultrasound imaging characteristics of the nodules, while the SWE was employed to measure their stiffness in both transverse and longitudinal thyroid scans. Twelve diagnostic patterns were formed by combining AmCAD diagnosis and SWE values, including isolation, series, parallel, and integration. The diagnostic performance was assessed using the receiver operating characteristic curve and area under the curve (AUC). Sensitivity, specificity, accuracy, missed malignancy rate, and unnecessary biopsy rate were also determined.

**Results:**

Various diagnostic schemes have shown specific advantages in terms of diagnostic performance. Overall, integrating AmCAD with SWE imaging in the transverse scan yielded the most favorable diagnostic performance, achieving an AUC of 72.2% (95% confidence interval (CI): 63.0–81.5%), outperforming other diagnostic schemes. Furthermore, in the subgroup analysis of nodules measuring <2 cm or 2–4 cm, the integrated scheme consistently exhibited promising diagnostic performance, with AUCs of 74.2% (95% CI: 61.9–86.4%) and 77.4% (95% CI: 59.4–95.3%) respectively, surpassing other diagnostic schemes. The integrated scheme also effectively addressed thyroid nodule management by reducing the missed malignancy rate to 9.5% and unnecessary biopsy rate to 22.2%.

**Conclusion:**

The integration of AmCAD and SWE imaging in the transverse thyroid scan significantly enhances the diagnostic performance for distinguishing benign and malignant thyroid nodules. This strategy offers clinicians the advantage of obtaining more accurate clinical diagnoses and making well-informed decisions regarding patient management.

## Introduction

Thyroid carcinoma, characterized as a spectrum of symptomatic or asymptomatic thyroid nodules, represents the predominant endocrine malignancy worldwide [1]. The prevalence of thyroid cancer has shown a noticeable upward trend, with a recent study demonstrating a gradual increase in morbidity rates by 32.4% in men and 13.1% in women between 2019 and 2030 [2, 3]. Additionally, a 2024 study indicates that thyroid cancer now ranks among the top five cancers in China, particularly among women, with incidence rates rising by 15.7% in females and 16.9% in males from 2000 to 2018 [4]. Furthermore, the incidence of thyroid cancer has also increased among adolescents, particularly those aged 15–19, with annual growth rates of 4–5% since 1998 [5]. This notable surge in thyroid cancer incidence can be primarily attributed to significant advancements in diagnostic imaging methodologies, characterized by enhanced sensitivity, as well as the increased utilization of fine needle aspiration cytology (FNAC) [6–8]. Despite the remarkable rise in global thyroid cancer incidence in the recent years, its impact on mortality rates has been minimal [9, 10]. Additionally, it is crucial to acknowledge that only a small fraction, approximately 10%, of thyroid nodules demonstrate malignancy [11]. This stark reality underscores the paramount importance of precise diagnosis for thyroid nodules, in order to avoid unnecessary overdiagnosis of benign nodules and futile invasive procedures, while also advocating for early detection of malignancy and prompt treatment.

Ultrasound is the preferred first-line diagnostic imaging modality for distinguishing between benign and malignant thyroid nodules [12]. Diagnosis through ultrasound is based on the morphological features of nodules, such as composition, echogenicity, calcification, margins, and size. To standardize the evaluation process and improve diagnostic accuracy, several risk stratification systems, known as Thyroid Imaging Reporting and Data System (TI-RADS), have been developed. These systems categorize thyroid nodules based on sonographic features and assign risk levels of malignancy. Among the most widely used TI-RADS are the American College of Radiology (ACR) TI-RADS, European (EU) TI-RADS, and American Thyroid Association (ATA) TI-RADS, each differing slightly in terms of classification criteria and diagnostic thresholds [13–15]. While these guidelines help to standardize nodule assessment and improve consistency across clinical settings, ultrasound evaluations remain operator-dependent, leading to potential variability in diagnostic accuracy [16, 17].

Recent studies have compared the diagnostic performance of various TI-RADS systems. For example, Borlea et al. conducted a comparative analysis of the ACR, EU, Horvath, and French TI-RADS—and identified considerable differences in diagnostic metrics [18]. The ACR and EU TI-RADS exhibited higher sensitivity but relatively lower specificity, whereas the Horvath TI-RADS was more accurate but had reduced sensitivity. The French TI-RADS, developed by Russ et al., demonstrated superior overall diagnostic performance but requires further clinical validation. A meta-analysis by Piticchio et al. further compared ACR, EU, and Korean TI-RADS (K-TIRADS) and identified key differences in risk categorization [19]. The ACR TI-RADS often classifies a higher proportion of nodules as moderate risk, reducing unnecessary FNAC but potentially underestimating high-risk cases. In contrast, K-TIRADS tends to classify a larger percentage of nodules as high-risk, making it suitable for clinical settings where aggressive intervention is necessary, though it may also lead to a higher false-positive rate. The EU TI-RADS offers a more balanced risk distribution but tends to classify more nodules into mild-risk categories. These comparative studies underscore the need for further research to optimize diagnostic tools and strategies for thyroid nodule assessment, ensuring that the most effective approach is utilized in clinical practice.

The exponential growth of biomedical data, coupled with rapid advancements in medical and information technology, has driven significant progress in medical imaging analysis and led to remarkable advancements in computer-aided diagnosis (CAD) [20, 21]. This innovative approach involves extracting multiple features from imaging data to enable quantitative assessment of diseases, particularly tumors, which effectively addresses the challenge of evaluating both benign and malignant lesions [22, 23]. AmCAD, an FDA-approved CAD software device, enables real-time classification of thyroid nodules according to diverse TI-RADS guidelines [24]. Previous studies have extensively evaluated the performance of AmCAD in distinguishing benign and malignant thyroid nodules. Despite AmCAD demonstrates a diagnostic efficiency which is comparable to physicians, its diagnostic performance remains limited [25–27]. Shear wave elastography (SWE), an advanced non-invasive imaging technique, has garnered increasing applications in clinical practice due to its comprehensive and quantitative assessment of tissue stiffness [28]. This innovative modality operates by inducing shear waves in tissues through acoustic radiation force pulses. By precisely quantifying the shear wave propagation speed or converting it into Young’s modulus, accurate evaluation of tissue physical properties become possible [29]. Extensive research has validated the clinical significance of SWE in distinguishing benign and malignant thyroid nodules [30, 31]. Moreover, the integration of SWE with grayscale ultrasound in multimodal investigation has yielded promising results, and significantly enhances the diagnostic accuracy beyond the use of grayscale ultrasound alone [32, 33]. Despite these advancements, the potential synergistic impact of combining SWE with AmCAD on diagnostic efficacy remains uncertain. Further research is needed to explore the possible benefits and limitations of such a combination.

Thus, in the present study, we intend to comprehensively evaluate the diagnostic efficiency of various diagnostic patterns through the combination of AmCAD and SWE. This was accomplished by employing a range of diagnostic strategies, including (1) utilizing AmCAD and SWE independently, (2) combining them in parallel, (3) combining them sequentially, and (4) combining them in integration. The study protocol also encompassed the assessment of thyroid nodule elasticity obtained from both transverse and longitudinal ultrasound scans, as well as the consideration of diverse sizes of thyroid nodules. To the best of our knowledge, this investigation represents the first exploration of distinct diagnostic schemes that leverage AmCAD and SWE for thyroid nodule differentiation.

## Material and methods

### Ethics and consent

This prospective cross-sectional study was conducted at a single center, adhering to the principles outlined in the Declaration of Helsinki. The research study was approved by the Institutional Review Board of our institution (Reference number: HSEARS20190123004), and written consent was obtained from each participating patient.

### Study population

During the period of May 2019 to August 2021, patients with thyroid nodules were prospectively and consecutively enrolled. Each patient underwent grayscale ultrasound and two-dimensional shear wave elastography (2D-SWE) examinations, followed by preoperative FNAC and/or postoperative histopathological evaluation of the thyroid nodules. The inclusion criteria were as follows: (1) patients aged 18 years or older; (2) patients who underwent grayscale ultrasound and 2D-SWE examinations prior to FNAC and/or thyroidectomy; and (3) confirmation of thyroid nodule status by FNAC or histopathology. The exclusion criteria included: (1) patients with a history of thyroidectomy; (2) nodules with indeterminate or nondiagnostic results; and (3) cases with poor image quality. In situations involving multiple nodules within the same thyroid lobe, the nodule exhibiting the highest suspicion of malignancy or the largest nodule observed on ultrasound was chosen for subsequent analysis. The patient recruitment process was conducted by an independent, experienced thyroid surgeon who did not participate in any subsequent ultrasound examinations, image acquisition, or data analysis, thereby ensuring objectivity and minimizing potential biases in the study.

### Grayscale ultrasound and 2D-SWE examinations

Patients underwent grayscale ultrasound and 2D-SWE examinations in the week preceding FNAC or surgical procedures. All ultrasound imaging assessments were performed independently by a single sonographer with over three years of experience in thyroid ultrasound imaging. To ensure impartial and unbiased interpretations, the sonographer remained blind to the patients’ clinical information. All thyroid ultrasound examinations were performed using the Aixplorer Ultrasound imaging system (SuperSonic Imagine, Aix-en-Provence, France) equipped with a linear array probe (SL15-4, 4–15 MHz).

**Fig. 1:**
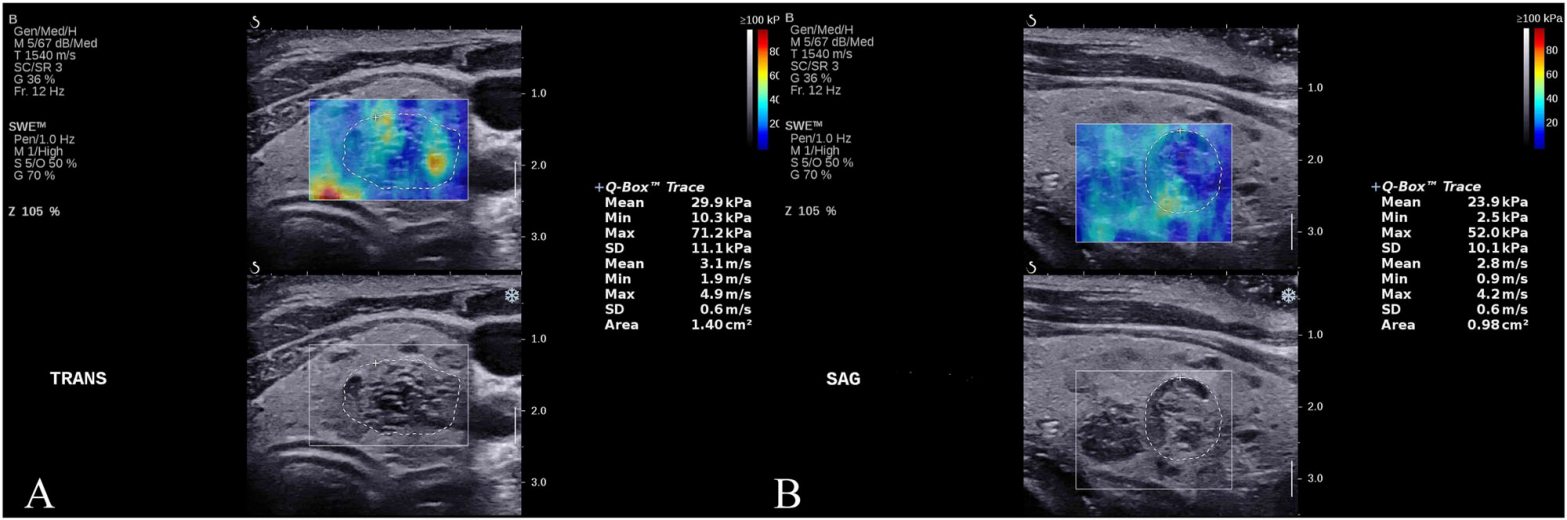
Representative shear wave elastography images of a thyroid nodule, presented in both transverse section (**A**) and longitudinal section (**B**). The elastography procedure employs the Q-box, guided in real-time by B-mode ultrasound, to trace the outline of the nodule. Simultaneously, the elastic value of the nodule is automatically calculated and displayed on the right side of the screen

Grayscale ultrasound was first used to measure the largest diameter of the thyroid nodule. The transverse scan showing the largest cross-sectional area of the nodule was saved for further analysis. Real-time 2D-SWE was then performed, with the sonographer positioning a region of interest (ROI) that included the entire thyroid nodule and surrounding tissue. The inbuilt quantification tool (Q-Box™) was utilized to calculate the mean elasticity of the nodule, excluding cystic areas, calcifications, and regions without color coding (Fig. 1). To ensure high measurement accuracy, the SWE measurements were conducted at three different transverse (referred to as SWE_T_) and longitudinal (referred to as SWE_L_) images of the nodule, respectively. The arithmetic mean of the three measurements was considered as the nodule’s elasticity at the corresponding scan plane.

Detailed procedural information for grayscale ultrasound and 2D-SWE examinations is available in the supplementary materials.

### CAD analysis

**Fig. 2:**
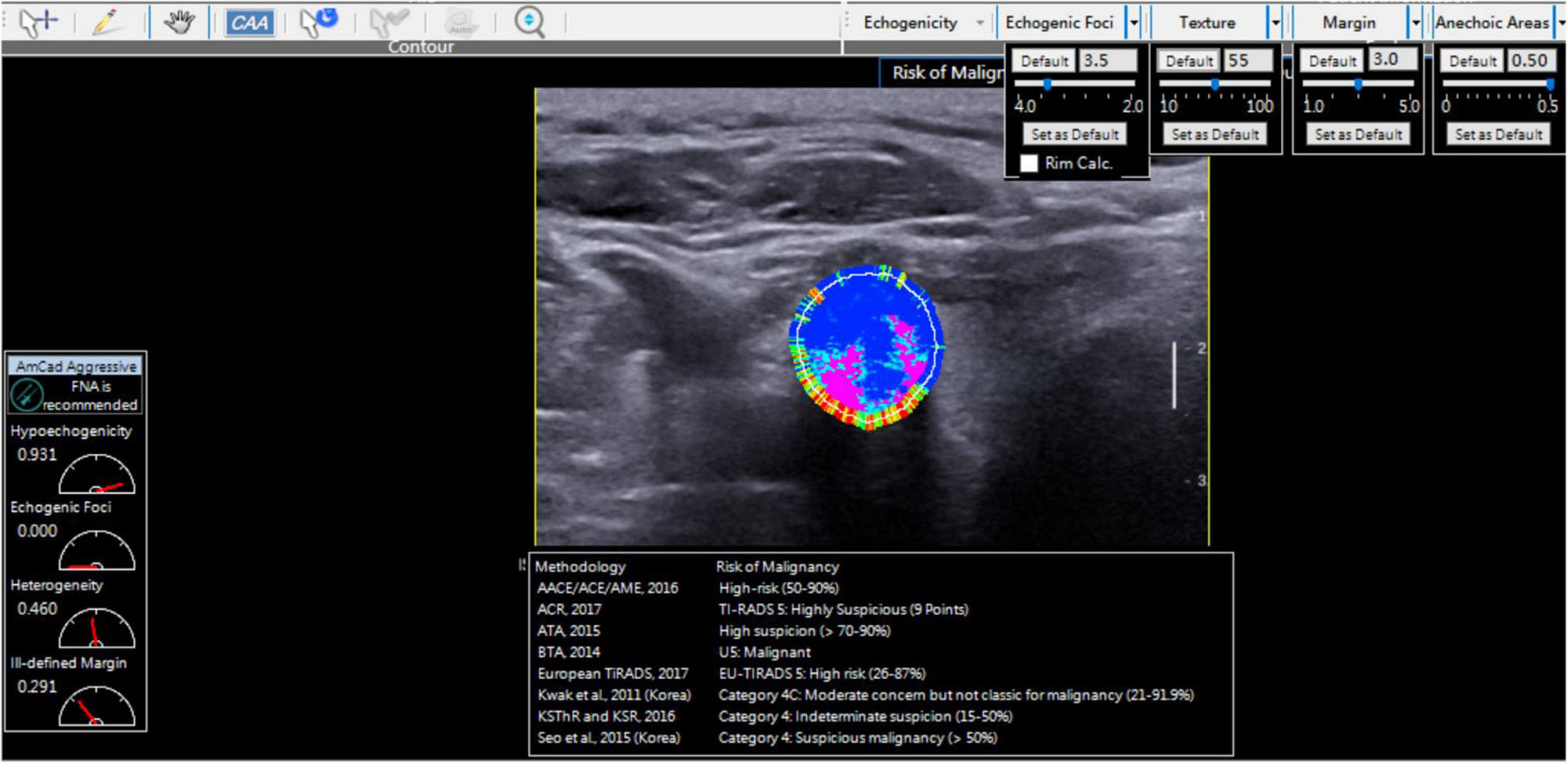
Interactive interface of the AmCAD diagnosis system, demonstrating its automated identification and precise quantification of ultrasound attributes, while also providing an accurate assessment of TI-RADS grade in thyroid nodule

After the data collection, the same sonographer performed the CAD analysis of the grayscale ultrasound images using the AmCAD software (AmCAD BioMed Corp., Taipei, Taiwan, China) following one month of training in the software. The transverse scan image of the thyroid nodule was imported into AmCAD software for analysis. In the image analysis, AmCAD software automatically delineated the nodule boundary, establishes an ROI, and extracted ultrasound-based morphological characteristics (Fig. 2). These distinctive features, including echogenicity, echogenic foci, margin, tumor shape, taller-than-wider configuration, texture, and composition, were identified and quantified using a diverse array of color-coded parameters.

Leveraging these discernible traits, AmCAD software generated a comprehensive report of evaluating malignancy risk and providing recommendations for thyroid nodules following eight TI-RADS guidelines. Given the findings from our prior investigation, wherein the EU-TIRADS exhibited better diagnostic performance than other TI-RADS guidelines [24], the present study adopted the EU-TIRADS guideline as the standardized approach in the image evaluation.

### Diagnostic strategy

The data analysis was independently performed by a radiologist with more than 5 years of experience in the field. The present study established twelve distinct diagnostic schemes as outlined below:

1. Isolated utilization of AmCAD;
2. Sole employment of SWE_T_;
3. Exclusive implementation of SWE_L_;
4. Parallel amalgamation of AmCAD with SWE_T_;
5. Parallel amalgamation of AmCAD with SWE_L_;
6. Parallel amalgamation of AmCAD, SWE_T_, and SWE_L_;
7. Serial fusion of AmCAD with SWE_T_;
8. Serial fusion of AmCAD with SWE_L_;
9. Serial fusion of AmCAD, SWE_T_, and SWE_L_;
10. Integration employment of AmCAD with SWE_T_;
11. Integration employment of AmCAD with SWE_L_;
12. Integration employment of AmCAD, SWE_T_, and SWE_L_.

In the isolated scheme, AmCAD, SWE_T_, or SWE_L_ were used individually to differentiate between benign and malignant thyroid nodules. The parallel strategy classified a thyroid nodule as malignant if any of the diagnostic variables indicated malignancy. In contrast, the serial strategy identified a thyroid nodule as malignant only when all diagnostic variables concurred with this classification. The integrated strategy utilized a logistic regression algorithm to incorporate all diagnostic variables for calculating the patient’s risk probability and provided diagnostic outcomes based on a pre-determined threshold.

### Statistical analysis

Data analyses were performed using SPSS 26.0 software (SPSS Inc., Chicago, IL, USA) and R statistical software (version 4.2.0; http://www.R-project.org). Continuous variables were presented as mean ± standard deviation or as median (interquartile range), while categorical variables were reported as frequencies (percentage). The receiver operating characteristic (ROC) curve was constructed to evaluate the performance of different diagnostic schemes in differentiating between benign and malignant thyroid nodules, with pathology results serving as the reference standard. The area under the ROC curve (AUC) was calculated to measure performance, and a *Delong* test was used to compare AUCs for different diagnostic schemes. The *DeLong* test was chosen because it is a non-parametric method specifically designed to compare the AUCs of correlated ROC curves, which is the case in our study since the same set of nodules is being evaluated by different diagnostic schemes. This method accounts for the paired nature of the data, providing a robust and unbiased comparison of the diagnostic performances. The Youden index, derived from ROC analysis, was employed to identify the optimal cutoff value for the diagnostic schemes by locating the point of maximum tangent. Additionally, sensitivity, specificity, accuracy, missed malignancy rate, and unnecessary biopsy rate were determined. The missed malignancy rate represents the proportion of nodules identified as benign despite being malignant among currently total biopsy-required nodules. The unnecessary biopsy rate is defined as the proportion of misdiagnosed benign nodules among the currently total biopsy-required nodules. Furthermore, the overall rate, which is the average of the sum of the missed malignancy rate and unnecessary biopsy rate, was also calculated. To assess the impact of thyroid nodule size on the diagnostic strategy, a subgroup analysis was conducted based on the Tumor, node and metastasis (TNM) staging for thyroid cancer, categorizing the nodules into two distinct groups: those with a size smaller than 2 cm and those measuring between 2 cm and 4 cm [34]. Statistical significance was defined as a two-sided *P* value of less than 0.05.

## Result

### Baseline characteristics of study population

A total of 126 thyroid nodules involving 122 patients were included in this study. Among these nodules, 80 (63.5%) were pathologically confirmed as benign, while 46 (36.5%) were identified as malignant. The size of benign nodules ranged from 0.53 cm to 5.25 cm, with an average size of 2.55 ± 1.21 cm. Conversely, the malignant nodules ranged from 0.57 cm to 5.63 cm, with an average size of 1.89 ± 1.28 cm. Based on their size, the nodules were distributed as follows: 64 nodules (50.8%) were smaller than 2 cm, 48 nodules (38.1%) fell within the 2 to 4 cm range, and 14 nodules (11.1%) exceeded 4 cm in size. The benign nodules exhibited a SWE_T_ value of 14.40 kPa (9.80–18.88 kPa), significantly lower than the SWE_T_ value found in malignant nodules, which measured 18.81 kPa (11.85–30.10 kPa) (*P* = 0.009). Similarly, the SWE_L_ value for benign nodules was 16.47 kPa (11.64–22.71 kPa), demonstrating a lesser measurement compared to malignant nodules, which recorded 19.87 kPa (14.26–36.71 kPa) (*P* = 0.027). Detailed baseline characteristics of the study cohort are presented in Table 1.

**Table 1:** Baseline characteristics of patients and thyroid nodules

Characteristic	Total	Benign	Malignant
Patients	122	80	42
Sex (Male/Female)	21/101	14/66	7/35
Age (years)	54.01 ± 12.13	53.81 ± 12.21	54.38 ± 12.10
Masses	126	80 (63.5)	46 (36.5)
Maximum diameter (cm)	2.31 ± 1.27	2.55 ± 1.21	1.89 ± 1.28
range (cm)	0.53–5.63	0.53–5.25	0.57–5.63
<2 cm	64	31 (48.4)	33 (51. 6)
2–4 cm	48	39 (81.2)	9 (18.8)
>4 cm	14	10 (71.4)	4 (28.6)
AmCAD			
EU TI-RADS 2	4	2 (50.0)	2 (50.0)
EU TI-RADS 3	31	27 (87.1)	4 (12.9)
EU TI-RADS 4	16	12 (75.0)	4 (25.0)
EU TI-RADS 5	75	39 (52.0)	36 (48.0)
SWE_T_ value (kPa)	15.08 (10.30–22.37)	14.40 (9.80–18.88)	18.81 (11.85–30.10)
SWE_L_ value (kPa)	17.89 (12.62–24.84)	16.47 (11.64–22.71)	19.87 (14.26–36.71)

Categorical variables are presented as n (%) and continuous variables as mean ± standard deviation or median (interquartile range), as appropriate. SWE_T_ indicates elastic value measures in the transverse section. SWE_L_ indicates elastic value measures in the longitudinal section

*SWE* shear wave elastography

### Performance of different diagnosis strategies

#### Optimal thresholds

The optimal threshold was established based on the Youden index, resulting in the designation of EU-TIRADS 5 for AmCAD, and the cutoff values of 19.28 kPa for SWE_T_ and 28.2 kPa for SWE_L_, respectively.

#### AUC

**Fig. 3:**
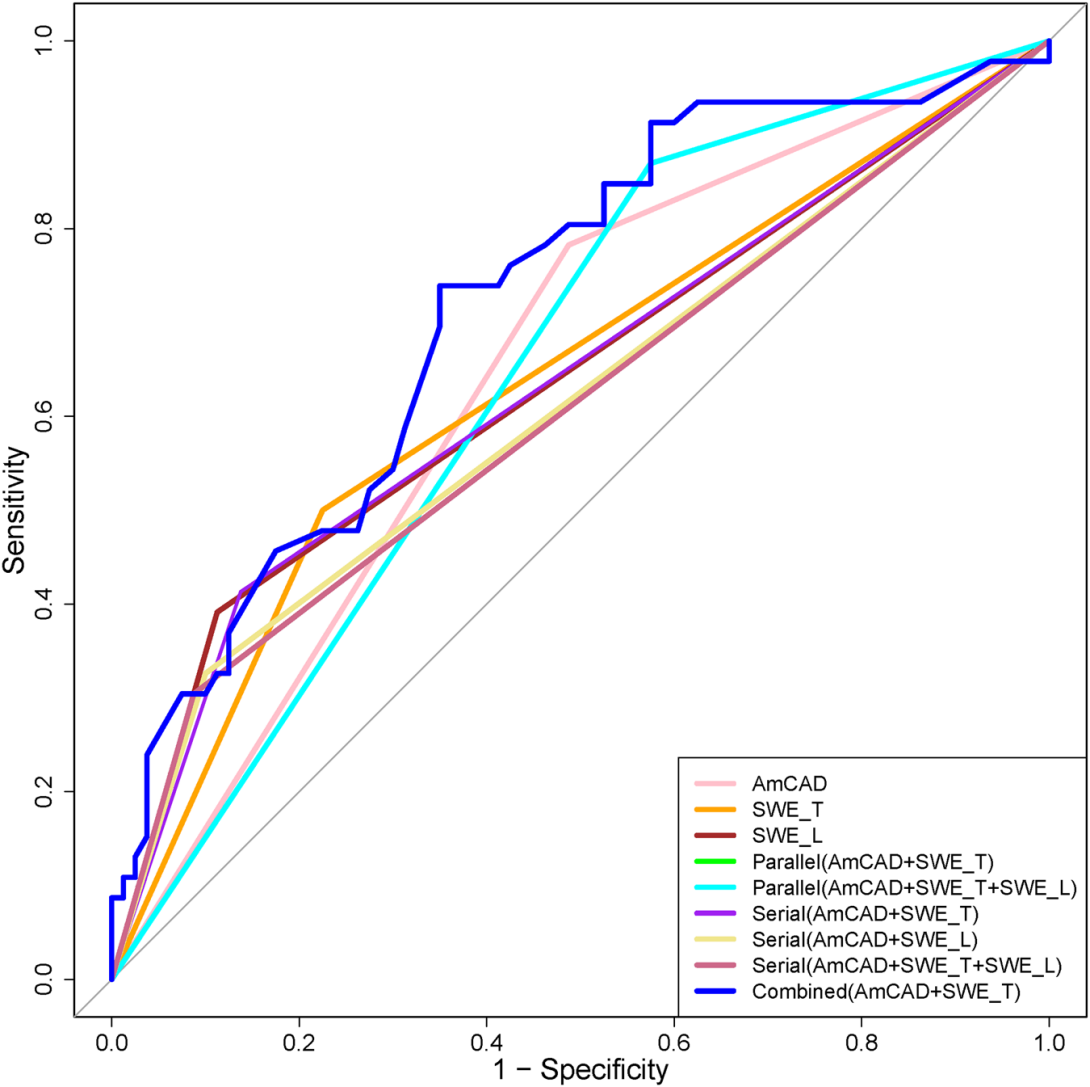
Comparison of receiver operating character curves for the leading diagnostic strategies

The integrated scheme combining AmCAD and SWE_T_ demonstrated significant diagnostic efficacy, with an AUC of 72.2% (95% confidence interval (CI): 63.0–81.5%). This performance significantly exceeded that of other diagnostic schemes (*P* values: 0.004–0.054), except for the parallel scheme (AmCAD + SWE_L_) and alternative integrated schemes, which showed AUCs ranging from 67.4 to 72.1% (*P* values: 0.136–0.856) (Fig. 3).

#### Sensitivity and specificity

Both parallel schemes, AmCAD+SWE_T_ and AmCAD+SWE_T_ + SWE_L_, achieved identical and notable sensitivity of 87.0% (95% CI: 73.7–95.1%), while the serial scheme (AmCAD+SWE_T_ + SWE_L_) yielded superior specificity of 91.3% (95% CI: 82.8–96.4%).

#### Diagnostic accuracy

Among all diagnostic strategies, SWE_L_ demonstrated the highest diagnostic accuracy of 70.6% (95% CI: 61.9–78.4%).

**Fig. 4:**
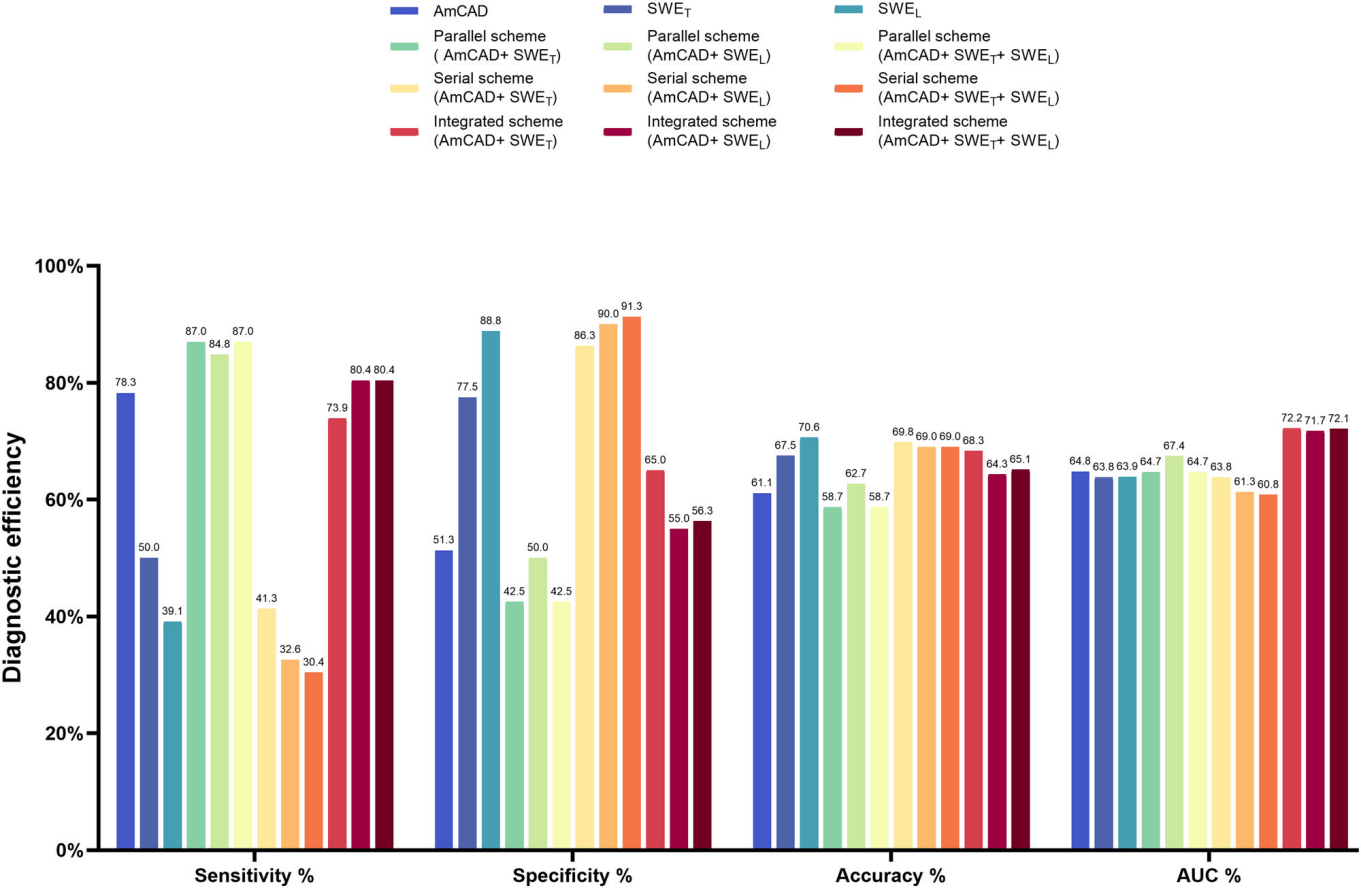
A comparison of diagnostic metrics for each diagnostic strategy

The performance of all the diagnosis strategies is detailed in Table 2 and Fig. 4.

**Table 2:** Comparison of diagnostic performance between various schemes

Index	Sensitivity % (95% CI)	Specificity % (95% CI)	Accuracy % (95% CI)	AUC % (95% CI)	*P* value
AmCAD	78.3 (63.6–89.1)	51.3 (39.8–62.6)	61.1 (52.0–69.7)	64.8 (55.0–74.6)	0.026
SWE_T_ modality	50.0 (34.9–65.1)	77.5 (66.8–86.1)	67.5 (58.5–75.5)	63.8 (53.4–74.1)	0.041
SWE_L_ modality	39.1 (25.1–54.6)	88.8 (79.7–94.7)	**70.6 (61.9**–**78.4)**	63.9 (53.4–74.4)	0.054
Parallel scheme (AmCAD+ SWE_T_)	**87.0 (73.7**–**95.1)**	42.5 (31.5–54.1)	58.7 (49.6–67.4)	64.7 (55.1–74.4)	0.024
Parallel scheme (AmCAD+ SWE_L_)	84.8 (71.1–93.7)	50.0 (38.6–61.4)	62.7 (53.6–71.2)	67.4 (57.9–76.9)	0.136
Parallel scheme (AmCAD+ SWE_T_ + SWE_L_)	**87.0 (73.7**–**95.1)**	42.5 (31.5–54.1)	58.7 (49.6–67.4)	64.7 (55.1–74.4)	0.024
Serial scheme (AmCAD+ SWE_T_)	41.3 (27.0–56.8)	86.3 (76.7–92.9)	69.8 (61.0–77.7)	63.8 (53.3–74.2)	0.014
Serial scheme (AmCAD+ SWE_L_)	32.6 (19.5–48.0)	90.0 (81.2–95.6)	69.0 (60.2–77.0)	61.3 (50.7–71.9)	0.006
Serial scheme (AmCAD+ SWE_T_ + SWE_L_)	30.4 (17.7–45.8)	**91.3 (82.8**–**96.4)**	69.0 (60.2–76.7)	60.8 (50.2–71.5)	0.004
Integrated scheme (AmCAD+ SWE_T_)	73.9 (58.9–85.7)	65.0 (53.5–75.3)	68.3 (59.4–76.3)	**72.2 (63.0**–**81.5)**	**/**
Integrated scheme (AmCAD+ SWE_L_)	80.4 (66.1–90.6)	55.0 (43.5–66.2)	64.3 (55.3–72.6)	71.7 (62.4–81.0)	0.796
Integrated scheme (AmCAD+ SWE_T_ + SWE_L_)	80.4 (66.1–90.6)	56.3 (44.7–67.3)	65.1 (56.1–73.4)	72.1 (62.8–81.4)	0.856

SWE_T_ indicates elastic value measures in the transverse section. SWE_L_ indicates elastic value measures in the longitudinal section. *P* value indicates the comparison of AUCs between integrated scheme (AmCAD + SWE_T_) and other diagnostic strategies. The bold value signifies the highest diagnostic performance in this metric.

*SWE* shear wave elastography, *AUC* area under the curve, *CI* confidence interval.

### Performance of different diagnosis strategies in nodules size < 2 cm

#### AUC

When compared with other strategies, the three integrated schemes showed comparable and remarkable diagnostic performance, achieving AUCs ranging from 74.0 to 74.2%. These values exceeded those of all other schemes, which had AUCs varying from 58.8 to 66.3% (*P* values ranging from 0.004 to 0.165).

#### Sensitivity and specificity

Both parallel schemes, AmCAD+SWE_T_ and AmCAD+SWE_T_ + SWE_L_, achieved identical and optimal sensitivity of 97.0% (95% CI: 84.2–99.9%). The SWE_L_ scheme, together with the serial schemes, AmCAD+SWE_L_ and AmCAD+SWE_T_ + SWE_L_, attained the same highest specificity of 90.3% (95% CI: 74.3–98.0%).

#### Diagnostic accuracy

Among all schemes, the integrated scheme (AmCAD+SWE_T_) exhibited the highest accuracy of 73.4% (95% CI: 60.9–83.7%).

The performance of all diagnostic strategies is summarized in Table 3 and Fig. S1-2.

**Table 3:** Comparison of diagnostic performance between various schemes for nodule size <2 cm

Index	Sensitivity % (95% CI)	Specificity % (95% CI)	Accuracy % (95% CI)	AUC % (95% CI)	*P* value
AmCAD	87.9 (71.8–96.6)	38.7 (21.9–57.8)	64.1 (51.1–75.7)	63.3 (49.5–77.1)	0.062
SWE_T_ modality	45.5 (28.1–63.7)	77.4 (58.9–90.4)	60.9 (47.9–72.9)	61.4 (47.6–75.3)	0.032
SWE_L_ modality	36.4 (20.4–54.9)	**90.3** (**74.3**–**98.0)**	62.5 (49.5–74.3)	63.3 (49.7–77.0)	0.056
Parallel scheme (AmCAD+ SWE_T_)	**97.0** (**84.2**–**99.9)**	29.0 (14.2–48.0)	64.1 (51.1–75.7)	63.0 (49.1–76.9)	0.043
Parallel scheme (AmCAD+ SWE_L_)	93.9 (79.8–99.3)	38.7 (21.9–57.8)	67.2 (54.3–78.4)	66.3 (52.8–79.9)	0.165
Parallel scheme (AmCAD+ SWE_T_ + SWE_L_)	**97.0** (**84.2**–**99.9)**	29.0 (14.2–48.0)	64.1 (51.1–75.7)	63.0 (49.1–76.9)	0.043
Serial scheme (AmCAD+ SWE_T_)	36.4 (20.4–54.9)	87.1 (70.2–96.4)	60.9 (47.9–72.9)	61.7 (47.9–75.5)	0.011
Serial scheme (AmCAD+ SWE_L_)	30.3 (15.6–48.7)	**90.3** (**74.3**–**98.0)**	59.4 (46.4–71.5)	60.3 (46.4–74.2)	0.010
Serial scheme (AmCAD+ SWE_T_ + SWE_L_)	27.3 (13.3–45.5)	**90.3** (**74.3**–**98.0)**	57.8 (44.8–70.1)	58.8 (44.8–72.8)	0.004
Integrated scheme (AmCAD+ SWE_T_)	81.8 (64.5–93.0)	64.5 (45.4–80.8)	**73.4** (**60.9**–**83.7)**	**74.2** (**61.9**–**86.4)**	/
Integrated scheme (AmCAD+ SWE_L_)	90.9 (75.7–98.1)	48.4 (30.2–66.9)	70.3 (57.6–81.1)	74.0 (62.0–86.1)	0.971
Integrated scheme (AmCAD+ SWE_T_ + SWE_L_)	90.9 (75.7–98.1)	51.6 (33.1–69.9)	71.9 (59.2–82.4)	**74.2** (**62.1**–**86.3)**	> 0.999

SWE_T_ indicates elastic value measures in the transverse section. SWE_L_ indicates elastic value measures in the longitudinal section. *P* value indicates the comparison of AUCs between integrated scheme (AmCAD + SWE_T_) and other diagnostic strategies. The bold value signifies the highest diagnostic performance in this metric

*SWE* shear wave elastography, *AUC* area under the curve, *CI* confidence interval

### Performance of different diagnosis strategies in nodules size 2–4 cm

#### AUC

The integrated scheme, involving AmCAD and SWE_T_, exhibited notable diagnostic efficacy, supported by an AUC of 77.4% (95% CI: 59.4–95.3%), outperforming other diagnostic patterns (AUCs varying from 59.0 to 77.1%, *P* values ranging from < 0.001 to 0.944).

#### Sensitivity, specificity, and accuracy

The SWE_T_ scheme, as well as the parallel schemes, demonstrated the highest sensitivity of 77.8% (95% CI: 40.0–97.2%). The serial scheme (AmCAD+SWE_T_ + SWE_L_) showed the highest specificity of 89.7% (95% CI: 75.8–97.1%) and accuracy of 83.3% (95% CI: 69.8–92.5%).

An overview of the performance results for all diagnostic strategies is presented in Table 4 and Figs. S3–4.

**Table 4:** Comparison of diagnostic performance between various schemes for nodule size 2–4 cm

Index	Sensitivity % (95% CI)	Specificity % (95% CI)	Accuracy % (95% CI)	AUC % (95% CI)	*P* value
AmCAD	66.7 (29.9–92.5)	51.3 (34.8–67.6)	54.2 (39.2–68.6)	59.0 (38.5–79.4)	<0.001
SWE_T_ modality	**77.8** (**40.0**–**97.2)**	74.4 (57.9–87.0)	75.0 (60.4–86.4)	76.1 (58.3–93.8)	0.818
SWE_L_ modality	66.7 (29.9–92.5)	87.2 (72.6–95.7)	**83.3** (**69.8**–**92.5)**	76.9 (57.5–96.4)	0.944
Parallel scheme (AmCAD+ SWE_T_)	**77.8** (**40.0**–**97.2)**	43.6 (27.8–60.4)	50.0 (35.2–64.8)	60.7 (41.0–80.3)	0.003
Parallel scheme (AmCAD+ SWE_L_)	**77.8** (**40.0**–**97.2)**	51.3 (34.8–67.6)	56.3 (41.2–70.5)	64.5 (45.4–83.7)	0.023
Parallel scheme (AmCAD+ SWE_T_ + SWE_L_)	**77.8** (**40.0**–**97.2)**	43.6 (27.8–60.4)	50.0 (35.2–64.8)	60.7 (41.0–80.3)	0.003
Serial scheme (AmCAD+ SWE_T_)	66.7 (29.9–92.5)	82.1 (66.5–92.5)	79.2 (65.0–89.5)	74.4 (54.8–93.9)	0.321
Serial scheme (AmCAD+ SWE_L_)	55.6 (21.2–86.3)	87.2 (72.6–95.7)	81.3 (67.4–91.1)	71.4 (50.4–92.3)	0.298
Serial scheme (AmCAD+ SWE_T_ + SWE_L_)	55.6 (21.2–86.3)	**89.7** (**75.8**–**97.1)**	**83.3** (**69.8**–**92.5)**	72.6 (51.7–93.6)	0.406
Integrated scheme (AmCAD+ SWE_T_)	66.7 (29.9–92.5)	59.0 (42.1–74.4)	60.4 (45.3–74.2)	**77.4** (**59.4**–**95.3)**	**/**
Integrated scheme (AmCAD+ SWE_L_)	66.7 (29.9–92.5)	51.3 (34.8–67.6)	54.2 (39.2–68.6)	74.2 (56.2–92.3)	0.369
Integrated scheme (AmCAD+ SWE_T_ + SWE_L_)	66.7 (29.9–92.5)	51.3 (34.8–67.6)	54.2 (39.2–68.6)	77.1 (59.3–94.8)	0.780

SWE_T_ indicates elastic value measures in the transverse section. SWE_L_ indicates elastic value measures in the longitudinal section. *P* value indicates the comparison of AUCs between integrated scheme (AmCAD + SWE_T_) and other diagnostic strategies. The bold value signifies the highest diagnostic performance in this metric

*SWE* shear wave elastography, *AUC* area under the curve, *CI* confidence interval

### Performance of different diagnosis strategies in missed malignancy rate and unnecessary biopsy rate

#### Missed malignancy rate

Both parallel schemes, specifically AmCAD+SWE_T_ and AmCAD+SWE_T_ + SWE_L_, exhibited a consistent and minimal missed malignancy rate of 4.8%.

#### Unnecessary biopsy rate

The serial scheme (AmCAD+SWE_T_ + SWE_L_) demonstrated the lowest unnecessary biopsies rate at 5.6%.

#### Overall rate

The SWE_L_ scheme achieved the lowest overall rate of 14.7%, followed by the serial schemes ranging from 15.1 to 15.5%, and the integrated scheme (AmCAD+SWE_T_) with a rate of 15.9%.

**Fig. 5:**
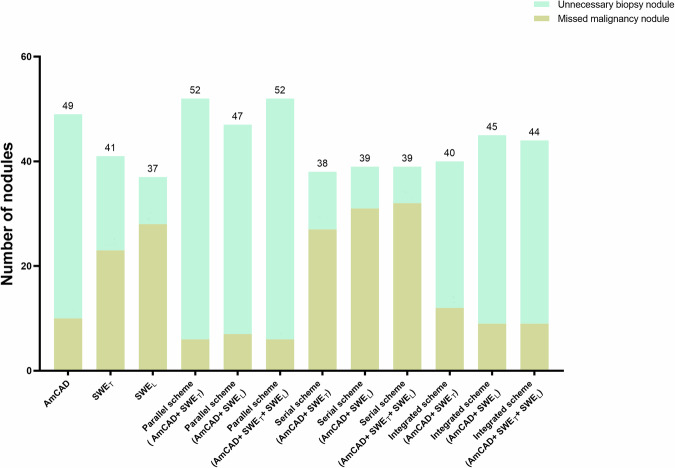
The numbers of missed malignancy nodules and unnecessary biopsy nodules obtained from each diagnostic strategy

Table 5 and Fig. 5 provide a comprehensive summary of the performance evaluation of various diagnostic strategies.

**Table 5:** Comparison of missed malignancy rate and unnecessary biopsy rate between various schemes

Index	Missed malignancy rate, %	Unnecessary biopsy rate, %	Overall rate, %
AmCAD	7.9 (10/126)	31.0 (39/126)	19.4
SWE_T_ modality	18.3 (23/126)	14.3 (18/126)	16.3
SWE_L_ modality	22.2 (28/126)	7.1 (9/126)	**14.7**
Parallel scheme (AmCAD+ SWE_T_)	**4.8** (**6/126)**	36.5 (46/126)	20.6
Parallel scheme (AmCAD+ SWE_L_)	5.6 (7/126)	31.7 (40/126)	18.7
Parallel scheme (AmCAD+ SWE_T_ + SWE_L_)	**4.8** (**6/126)**	36.5 (46/126)	20.6
Serial scheme (AmCAD+ SWE_T_)	21.4 (27/126)	8.7 (11/126)	15.1
Serial scheme (AmCAD+ SWE_L_)	24.6 (31/126)	6.3 (8/126)	15.5
Serial scheme (AmCAD+ SWE_T_ + SWE_L_)	25.4 (32/126)	**5.6** (**7/126)**	15.5
Integrated scheme (AmCAD+ SWE_T_)	9.5 (12/126)	22.2 (28/126)	15.9
Integrated scheme (AmCAD+ SWE_L_)	7.1 (9/126)	28.6 (36/126)	17.9
Integrated scheme (AmCAD+ SWE_T_ + SWE_L_)	7.1 (9/126)	27. 8 (35/126)	17.5

SWE_T_ indicates elastic value measures in the transverse section. SWE_L_ indicates elastic value measures in the longitudinal section. The bold value signifies the best performance in this metric

*SWE* shear wave elastography

## Discussion

In the present study, we leveraged a CAD device known as AmCAD in conjunction with the SWE technique to distinguish malignant thyroid nodules from benign nodules. Different diagnostic schemes were employed, utilizing a variety of combinations of these two techniques, including isolation, parallel utilization, serial implementation, and integration. In addition, the study assessed tissue elasticity of the thyroid nodules in both transverse and longitudinal scan images, as well as the impact of different nodule sizes on diagnostic strategies. The different diagnostic schemes demonstrated specific advantages in terms of diagnostic performance. Some schemes excelled in determining malignant nodules, while others showed proficiency in identifying benign nodules and reducing the rate of unnecessary biopsies. Additionally, the diagnostic performance of various strategies varied depending on the size of the nodule. The comprehensive evaluation of the present study revealed that the integrated scheme, in which AmCAD and SWE_T_ were combined, provided favorable diagnostic performance for discriminating between benign and malignant thyroid nodules, with an AUC of 72.2% (95% CI: 63.0–81.5%), exceeding other diagnostic strategies. Particularly, in the subcategories of nodules smaller than 2 cm and between 2 cm and 4 cm, the AUC values were 74.2% (95% CI: 61.9–86.4%) and 77.4% (95% CI: 59.4–95.3%), respectively, also showing promising diagnostic performance. Moreover, in the evaluation of missed malignancies and unnecessary biopsies, the integrated scheme yielded a favorable overall result of 15.9%.

CAD diagnostic system demonstrates its capability to perform ROI segmentation, feature extraction, and model construction. This is done by extracting a lot of relevant information from ultrasound images. Through the comprehensive mining and analysis of image data, the CAD system facilitates more accurate and efficient diagnoses, emerging as a novel diagnostic scheme in clinical practice [35, 36]. In this study, we employed the AmCAD detection system, which serves as an auxiliary diagnostic tool for thyroid ultrasound examination. This innovative system seamlessly integrates thyroid ultrasound image processing with nodule morphological characteristics analysis, offering valuable insights for accurate diagnosis. Previous studies exploring the implementation of AmCAD for diagnosing thyroid nodules have demonstrated comparable or superior diagnostic performance in comparison to junior radiologists. Nevertheless, the clinical utility has been limited, because of the low overall diagnostic accuracy with an AUC ranging from 58.5 to 74.8% [25, 26, 37]. These findings are consistent with our study in which AmCAD alone yielded a sensitivity of 78.3%, specificity of 51.3%, accuracy of 61.1%, and AUC of 64.8%. The limited diagnostic efficacy of AmCAD alone is primarily due to the fact that numerous grayscale ultrasound features were common and demonstrated in both benign and malignant nodules [11]. While AmCAD can potentially distinguish between benign and malignant nodules, it relies exclusively on morphological assessment and its diagnostic efficacy depends on the presence of distinct morphological features characteristic of benignity and malignancy [38, 39]. Consequently, despite artificial intelligence-based CAD software assistance, the differential diagnosis of thyroid nodules remains challenging.

Carcinogenesis is a multifaceted process involving changes in stiffness, morphological characteristics, and vascular dynamics [40]. Tissue elasticity, a fundamental parameter of physical property, undergoes significant influence from pathophysiological mechanisms. Malignant lesions often demonstrate increased stiffness, whereas benign lesions exhibit lower elasticity [28]. In the context of thyroid cancer, the increased stiffness can be attributed to fibroblast reactions, the presence of granular structures, and cellular compaction resulting from invasive cancer growth [41, 42]. The application of SWE imaging as an adjunct tool for evaluating thyroid nodule stiffness has emerged as a valuable biomarker for thyroid cancer [30]. Various investigations have demonstrated that the combined utilization of SWE and grayscale ultrasound, capitalizing on both the physical properties and morphological characteristics of thyroid nodules, enhances the diagnostic efficacy in detecting thyroid malignancies, surpassing the isolated use of grayscale ultrasound or SWE [32, 33, 43]. However, our study further contributes to the field by exploring the diagnostic variability based on nodule size and scanning orientation, providing a more nuanced understanding of the strengths and limitations of this integrated approach.

The SWE values obtained in thyroid nodules exhibited inconsistencies between transverse and longitudinal scans. Although the overall diagnostic performance was similar between the two, there were still noticeable differences. We hypothesize that these discrepancies are primarily due to two factors: First, the spatial heterogeneity of tumor tissue plays a crucial role in this phenomenon. Tumor cells exhibit variations in collagen fiber structure and density depending on their growth direction [44]. This biological complexity leads to different mechanical properties being displayed in different directions. Consequently, SWE measurements may yield different elasticity values depending on the scan plane. Specifically, certain regions of the tumor may appear stiffer in one direction while being softer in another. This spatial heterogeneity reflects the intricate internal structure of tumor tissues, where variations in fiber orientation and density can result in differing elasticity measurements between transverse and longitudinal scans [45]. Second, the anatomical differences in thyroid nodules between transverse and longitudinal planes, as well as the variation in acoustic push pulses, could also contribute to the inconsistency in elasticity measurements. SWE relies on the ultrasound probe emitting acoustic push pulses to excite the tissue. The anatomical structures surrounding the nodule (e.g., blood vessels, muscle tissues) and their relative positions to the ultrasound probe vary between different planes [46]. These variations can affect the propagation path and focusing of the sound waves, leading to inconsistent elasticity measurements. Specifically, during transverse and longitudinal scans, the probe’s position on the nodule may differ, causing variations in wave focusing, scattering, and attenuation, thereby impacting the measurement results [47]. While further clinical trials are needed to validate these hypotheses, exploring combined diagnostic strategies from different measurement planes is also essential.

This study employed a combination of AmCAD and SWE imaging to develop diverse diagnostic strategies, aiming to emphasize specific advantages of different combinations of these diagnostic tools. The parallel combination exhibited improved sensitivity but reduced specificity, while the serial combination offered enhanced specificity at the expense of sensitivity. Overall, the integration of AmCAD with SWE_T_ demonstrated the most favorable diagnostic performance, with an AUC of 72.2% (95% CI: 63.0–81.5%), significantly surpassing other combined diagnostic strategies. Moreover, in the subgroup analysis of thyroid nodules measuring <2 or 2–4 cm, the integration of AmCAD with SWE_T_ consistently showed promising diagnostic performance, with AUCs of 74.2% (95% CI: 61.9–86.4%) and 77.4% (95% CI: 59.4–95.3%) respectively, outperforming other diagnostic strategies. This integrated scheme also effectively addresses thyroid nodule management by reducing missed malignancy rates and unnecessary biopsy rates. By employing a combination of two assessment methods, the integrated strategy maximizes the analytical information obtained from available resources, thereby enhancing and optimizing diagnostic capabilities to support clinical decision-making [48]. Additionally, this strategy may be applicable to other areas of CAD-based ultrasound diagnostics, such as breast lesions [49], suggesting a broader impact on clinical practice beyond thyroid nodules.

Although this study has made noteworthy progress, it is important to acknowledge certain inherent limitations. First, the relatively small sample size in our study emphasizes the need for a more extensive prospective study on a larger scale to further substantiate and reinforce our findings. Second, the single-center design could introduce site-specific biases, such as differences in operator expertise or patient demographics, which may limit the generalizability of our results. A multicenter study would be needed to address these concerns and validate our findings across diverse clinical settings. Third, our study population consisted exclusively of patients undergoing thyroid biopsy or surgery, which may have introduced selection bias by excluding patients with less suspicious or indeterminate nodules. This could have affected the estimation of the diagnostic performance of the AmCAD software. Future research should include a broader spectrum of thyroid nodules to better reflect real-world clinical practice. Fourth, variability in ultrasound equipment and image resolution across different manufacturers could impact the diagnostic accuracy of the CAD software, highlighting the need for standardization and further investigation into the generalizability of the CAD system in different clinical environments.

## Conclusion

This study demonstrated the effectiveness of integrating AmCAD and SWE_T_ to enhance diagnostic performance in distinguishing between benign and malignant thyroid nodules. This approach offers significant potential for improving clinical diagnoses and decision-making. However, to confirm these findings and broaden their applicability, larger multi-center studies are needed. Future research should also evaluate the long-term impact of this diagnostic approach on clinical outcomes, including its role in reducing unnecessary biopsies and missed malignancies. Expanding the study population and assessing the diagnostic utility across diverse clinical settings will further elucidate its clinical value.

## Supplementary information

Supplementary Information

## Data Availability

The data presented in this study are available from the corresponding author upon reasonable request. Data is not publicly available due to privacy or ethical concerns.
