# Supplementary material for: Improving the diagnostic strategy for thyroid nodules: a combination of artificial intelligence-based computer-aided diagnosis system and shear wave elastography
Source: Endocrine. 2024 Oct 7;87(2):744–57. doi: 10.1007/s12020-024-04053-2 (PMC11811255; doi:10.1007/s12020-024-04053-2)
Supplement: Supplementary file 1 — Supplementary Information [file 12020_2024_4053_MOESM1_ESM.docx]

**Detailed Procedural Information for Grayscale Ultrasound and 2D-SWE Examinations**

Patients underwent grayscale ultrasound and 2D-SWE examinations in the week preceding FNAC or surgical procedures. All ultrasound imaging assessments were performed independently by a single sonographer with over three years of experience in thyroid ultrasound imaging. To ensure impartial and unbiased interpretations, the sonographer remained blind to the patients’ clinical information. All thyroid ultrasound examinations were performed using the Aixplorer Ultrasound imaging system (SuperSonic Imagine, Aix-en-Provence, France) equipped with a linear array probe (SL15-4, 4-15 MHz). Patients were positioned in a supine position with their neck hyperextended to facilitate complete exposure to the thyroid region.

Initially, conventional grayscale ultrasound was used to scan the thyroid nodule, and the largest diameter was recorded as the nodule size. The transverse scan demonstrating the largest cross-sectional area of the thyroid nodule was saved for further analysis. Subsequently, real-time 2D-SWE scan was performed with the transducer placed over the neck with minimal compression. Patients were instructed to take shallow breathing and avoid swallowing during the scanning. Real-time grayscale ultrasound, displayed in split-screen mode, guided the positioning of a rectangle-shaped region of interest (ROI) for 2D-SWE analysis, encompassing the entire thyroid nodule and its surrounding thyroid parenchyma.

To evaluate the tissue stiffness, the inbuilt quantification tool of the ultrasound system, Q-Box™, was employed to delineate the nodule’s contour, with efforts made to avoid intra-nodular cystic areas, calcifications, and areas lacking of SWE color coding. The mean elasticity within the Q-Box™ was automatically calculated and denoted as the “Mean” stiffness of the nodule. The aforementioned 2D-SWE examination was repeated within the longitudinal scan of the thyroid nodule.

To ensure high measurement accuracy, the SWE measurements were conducted at three different transverse (referred to as SWE_T_) and longitudinal (referred to as SWE_L_) images of the nodule, respectively. The arithmetic mean of the three measurements was considered as the nodule’s elasticity at the corresponding scan plane.


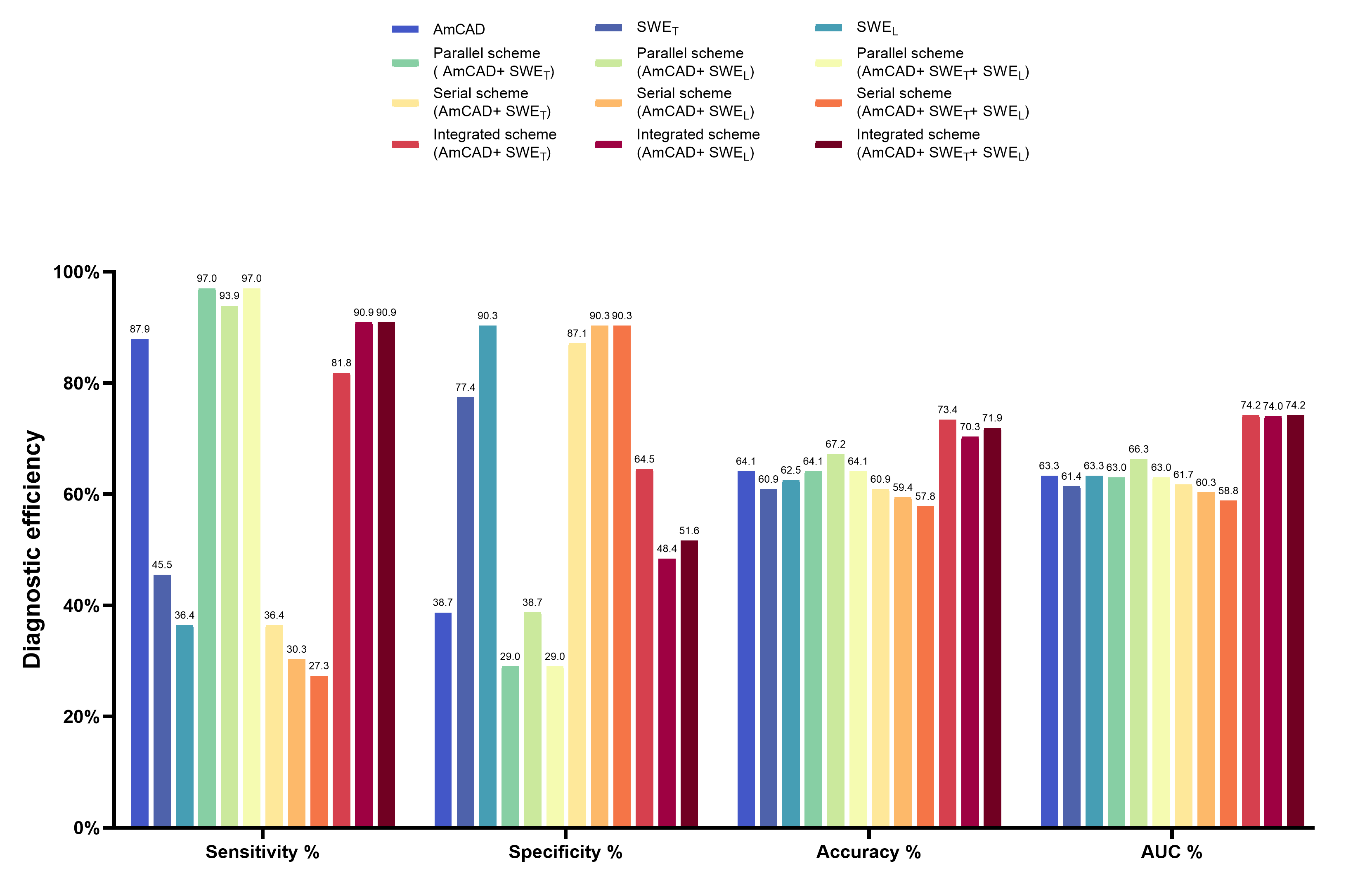


Figure S1. A comparison of diagnostic metrics for each diagnostic scheme in nodules size < 2 cm.


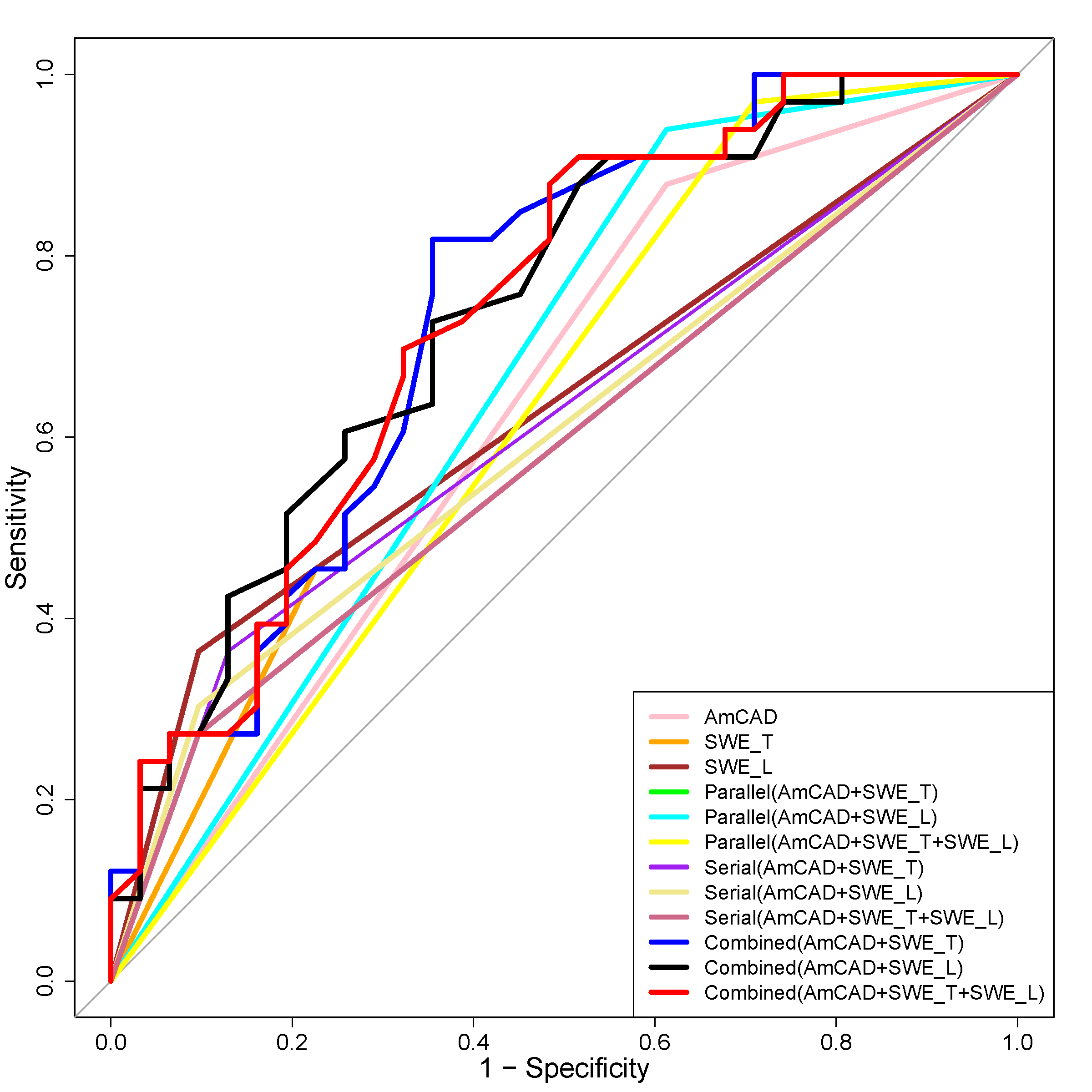


Figure S2. Comparison of receiver operating character curves for each diagnostic scheme in nodules size < 2 cm.


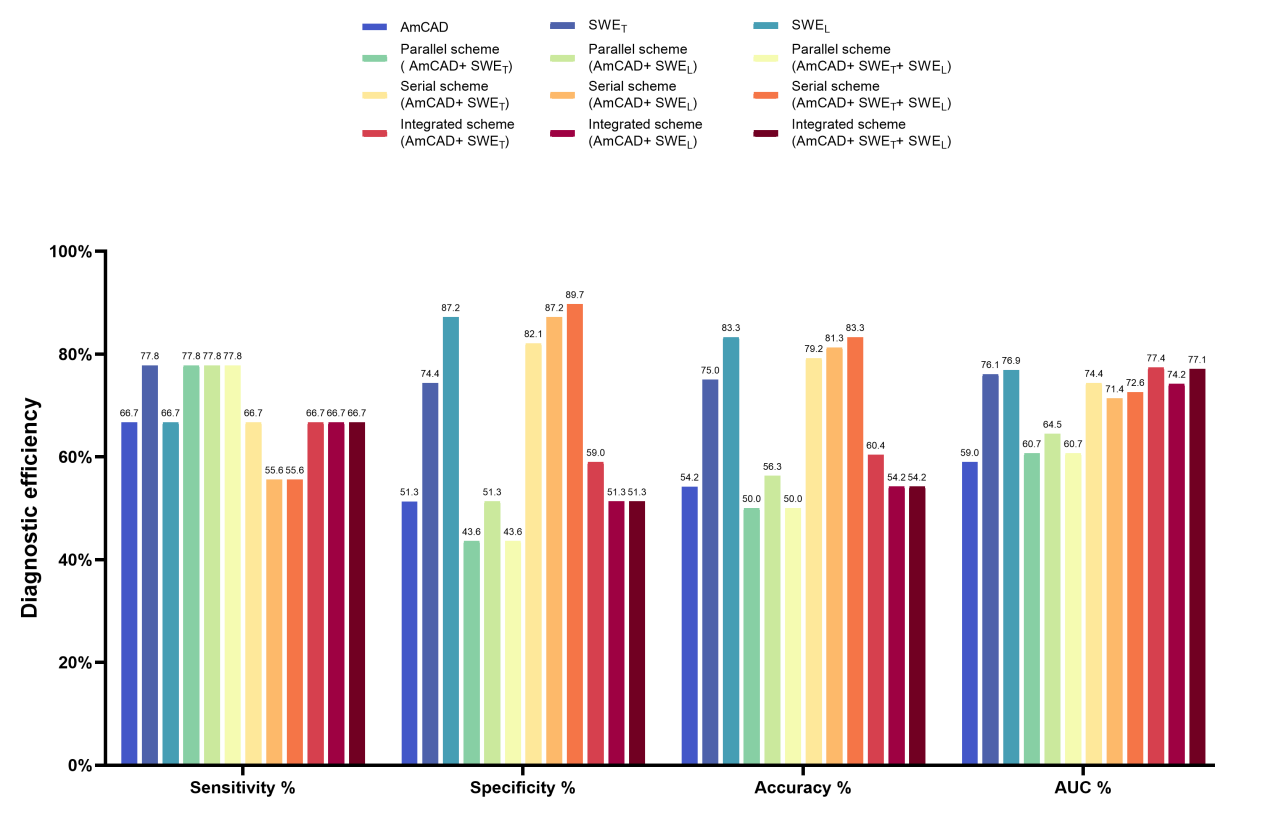


Figure S3. A comparison of diagnostic metrics for each diagnostic scheme in nodules size 2-4 cm.


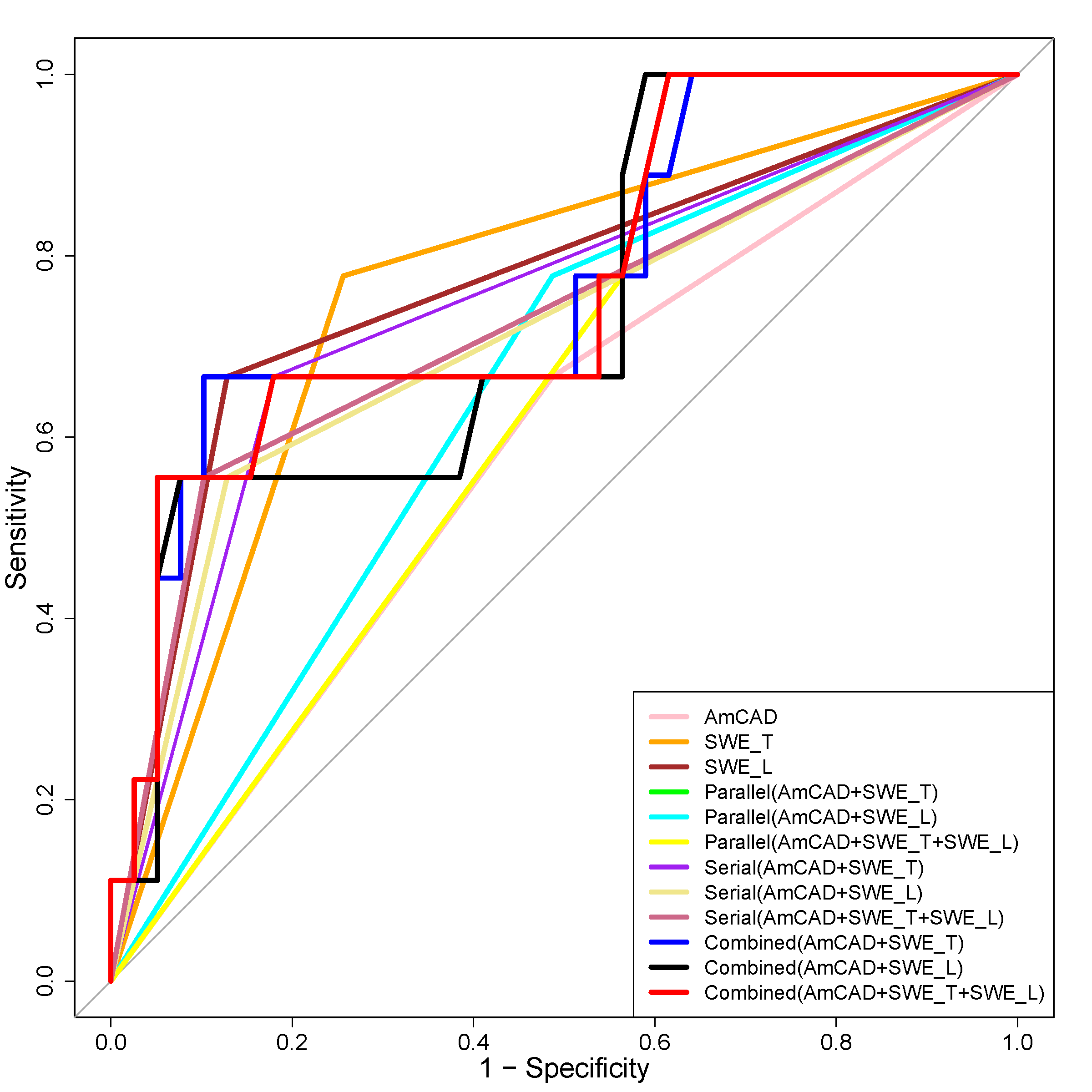


Figure S4. Comparison of receiver operating character curves for each diagnostic scheme in nodules size 2-4 cm.
